# Influence of fracture morphology and working length on shear motion and construct stiffness in osteosynthesis constructs

**DOI:** 10.1007/s00068-025-03011-5

**Published:** 2025-11-14

**Authors:** Marianne Hollensteiner, Mischa Mühling, Philipp Blum, Sabrina Sandriesser, Dirk Baumeister, Markus Greinwald, Julian Fürmetz, Peter Augat

**Affiliations:** 1 Institute for Biomechanics, BG Unfallklinik Murnau, Prof. Küntscher Str. 8, 82418 Murnau, Germany; 2 Institute for Biomechanics, Paracelsus Medical University Salzburg, Strubergasse 21, 5020 Salzburg, Austria; 3 Department of Trauma Surgery, BG Unfallklinik Murnau, Prof. Küntscher Str. 8, 82418 Murnau, Germany; 4 Department of Orthopaedics and Trauma Surgery, Musculoskeletal University Center Munich (MUM), University Hospital, LMU Munich, Marchioninistraße 15 81377 Munich, Germany; 5 Institute for Biomechanics, BG Unfallklinik Murnau and Paracelsus Medical University Salzburg, Prof. Küntscher Str. 8, 82418 Murnau, Germany

**Keywords:** Fracture morphology, Working length, Distal femur, Osteosynthesis, Torsional stiffness, Axial stiffness, Interfragmentary motion, Shear displacement

## Abstract

**Background:**

Construct stability is a key factor in fracture healing and is influenced by fracture morphology, working length, and fixation strategy. While osteotomized fracture models are widely used for biomechanical testing, their relevance to real, interdigitated fracture patterns remains unclear.

**Methods:**

This study compared the axial stiffness, torsional stiffness, and interfragmentary shear motion of synthetic distal femur models with osteotomized and realistic fractures. All constructs were tested under axial and torsional loading while progressively reducing the number of diaphyseal screws from five to two, thereby increasing the working length. Realistic fractures with a gap were analyzed in both an “open” state (prior to contact) and a “contact” state (after fragment contact. Shear displacements were quantified as resultant vectors derived from 3D motion tracking.

**Results:**

Fracture morphology and screw number significantly affected construct stiffness and shear motion. Osteotomized fractures showed higher axial stiffness (up to 997 N/mm in OC) compared to realistic fractures (up to 792 N/mm in RC), while realistic fractures without a gap exhibited superior torsional stability (up to 7.4 Nm/° in RC). Increasing working length reduced axial stiffness by up to -24% and torsional stiffness by up to -51%. Shear displacement increased with reduced screw number, particularly in constructs with a fracture gap.

**Conclusion:**

Realistic fractures exhibit complex and direction-dependent stabilization mechanisms that are not captured by osteotomized models. Working length strongly influences construct behavior across all configurations. This study highlights the biomechanical differences between osteotomized and realistic fractures. Osteotomized models remain valuable as reproducible worst-case scenarios, whereas realistic fractures provide complementary insights by capturing stabilizing mechanisms such as fragment interlocking. Both approaches should therefore be combined in biomechanical research. Clinically, the results underline the importance of anatomical reduction and fixation planning to maximize construct stability.

## Introduction

The biomechanical stability of osteosynthesis constructs is a critical determinant of fracture healing success. It directly influences the clinical outcome by ensuring sufficient stabilization to prevent implant failure, while also accommodating the biological processes necessary for bone repair. Among the key factors governing construct stability, the working length of osteosynthesis plates plays a pivotal role. Defined as the distance between the two screws closest to either side of the fracture, working length determines the stiffness, strain distribution, and fatigue resistance of the plate under load [1]. Shorter working lengths are characterized by increased stiffness, reduced plate strain, and enhanced construct stability [2, 3], minimizing the risk of implant failure. Conversely, longer working lengths allow for controlled flexibility, which can be beneficial in promoting secondary bone healing through callus formation in specific clinical scenarios [3, 4]. A critical determinant of fracture healing is the shear motion of fracture fragments, which is influenced by the working length [5]. Excessive shear motion can destabilize the fracture site, disrupting the biological environment necessary for healing. In contrast, controlled micromotion, within physiological limits, can stimulate callus formation and facilitate secondary bone healing [6, 7].

Despite the substantial body of research investigating these mechanical relationships in osteotomized fractures with planar surfaces [8–10], there is a relative paucity of data on the effects of working length on real fractures with irregular, interdigitated surfaces. Osteotomized models are widely used in biomechanical research due to their simplicity and reproducibility [11, 12]. These models, created by controlled cuts, produce flat, smooth fracture surfaces that allow consistent and repeatable testing conditions. However, these idealized fractures differ fundamentally from the interdigitated, irregular morphology of realistic fractures commonly encountered in clinical practice. Realistic fractures often provide inherent stability through fragment interlocking, which can reduce the mechanical demands on the osteosynthesis plate [13]. Moreover, the size of fracture gap significantly influences load distribution, strain, and micromotion [14, 15]. Open gaps increase reliance on the plate as the primary load-bearing element, raising the risk of implant fatigue and failure. Conversely, closed gaps enable load-sharing between bone and plate, reducing plate strain and enhancing stability [1, 3].

The interplay between axial and torsional loads further complicates the mechanical behavior of these constructs under dynamic physiological conditions, such as walking. Axial loads arising from weight-bearing interact with torsional moments generated during rotational movements, producing complex loading scenarios that challenge construct stability. In vivo telemetric implant measurements by Bergmann and colleagues demonstrated that such combined axial and torsional loads occur physiologically in the femur [16]. While static loading tests have provided valuable insights, the interaction between real fracture morphologies and combined loading conditions requires further investigation [17, 18]. Understanding how fracture morphology, gap size, and working length interact under these conditions is crucial for optimizing osteosynthesis strategies in clinical practice. These parameters collectively determine construct stiffness, interfragmentary motion, and the risk of secondary displacement, which in turn influence the mechanical environment for bone healing [19, 20]. Insufficient stability may lead to loss of reduction or implant failure, whereas excessive stiffness can impair callus formation and delay union [20]. By systematically analyzing these factors under physiologically relevant load scenarios, biomechanical studies can provide evidence-based recommendations for implant selection, working length configuration, and the need for additional fixation [21, 22].

This study addresses the existing knowledge gaps, specifically the lack of research on the biomechanical effects of working length and fracture gap size in realistic fractures with interdigitated morphology compared to osteotomized fractures with planar surfaces. While numerous studies have analyzed the mechanical properties of osteosynthesis constructs in idealized, osteotomized models, there is a lack of data on the stability and load distribution in realistic fractures with irregular fragment structures. Additionally, it remains insufficiently explored how different working lengths and fracture gap sizes influence interfragmentary motion and overall construct stiffness under varying loading conditions, particularly under axial and torsional loads. Therefore, this study specifically investigates the impact of these parameters on the biomechanical performance of both fracture types across varying loading scenarios. Although axial and torsional loading alone cannot replicate the full complexity of physiological joint loading, these simplified modes were selected as standardized conditions that allow systematic comparison between osteotomized and realistic fracture morphologies. This approach isolates the effect of fracture surface geometry and working length on construct mechanics, thereby complementing the more comprehensive but less controlled loading scenarios used in cadaveric models.

It is hypothesized that the morphology of fracture surfaces substantially influences construct mechanics beyond the effect of screw configuration alone. Specifically, we expected that realistic, interdigitated fracture surfaces would exhibit direction-dependent stabilization, leading to reduced interfragmentary motion and increased torsional stiffness compared to osteotomized fractures with planar surfaces. The realistic fracture model employed here reflects a patient-derived interdigitated morphology without comminution; other realistic fracture types, such as multi-fragmentary patterns, may demonstrate very different, less stable behaviors.

## Materials and methods

### Specimens

**Fig. 1:**
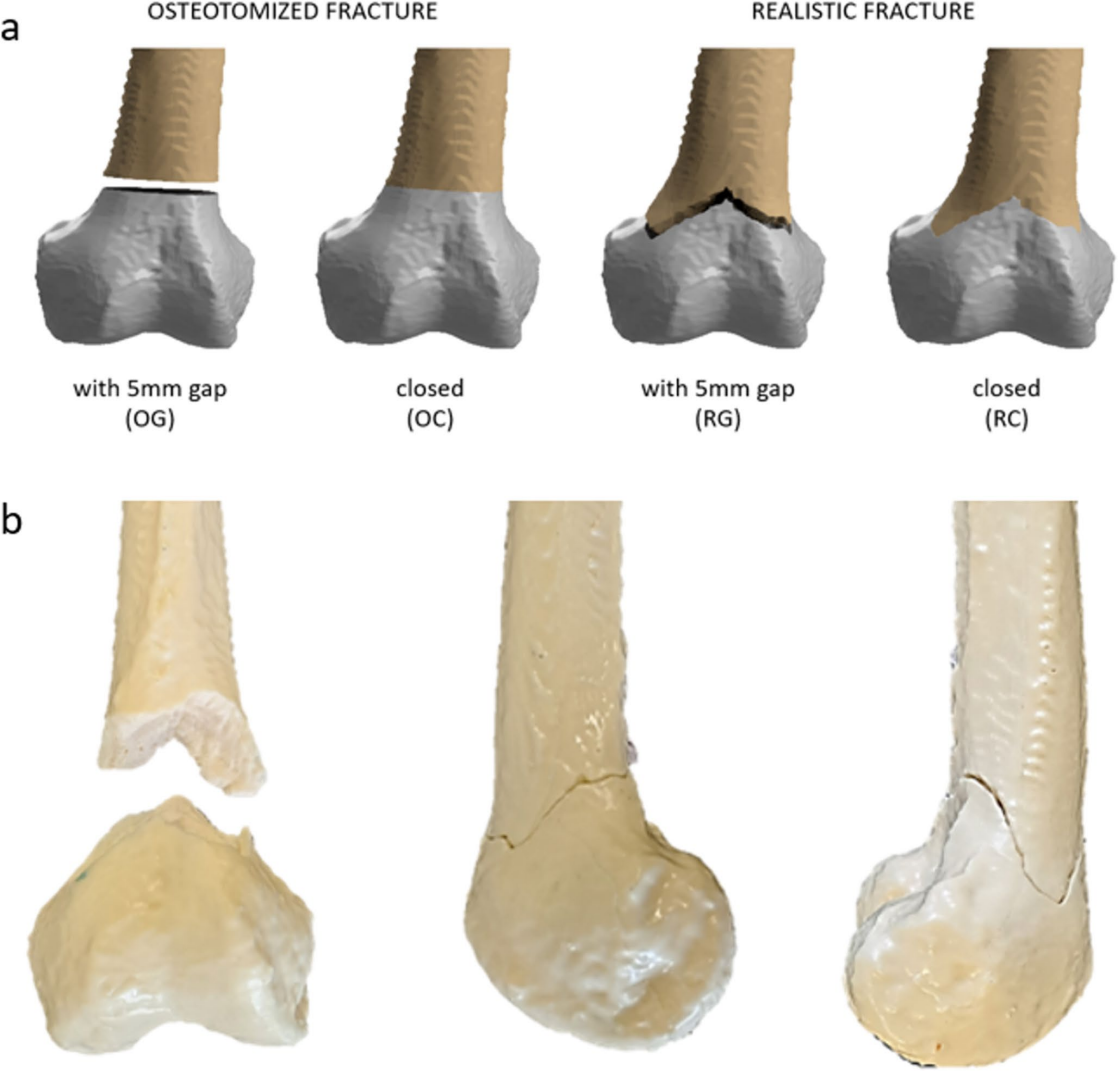
**a**: Illustration of the four femur groups: OG (osteotomized femur with a 5 mm gap), OC (osteotomized femur without a gap), RG (realistically fractured femur with a 5 mm gap), and RC (realistically fractured femur without a gap). **b**: Synthetic femur model with a realistic, interdigitated fracture morphology shown from anterior, lateral, and medial perspectives highlighting the complex geometry of the fracture surface

Synthetic distal femora models composed of polyurethane-based bone surrogates were used in this study; they included a cortical shell and an internal trabecular structure with a medullary canal, which have been morphologically and mechanically validated against human reference femora [23, 24]. Realistic fractures were derived from patient-specific CT scans, while osteotomized fractures were created using an oscillating saw to produce uniform and reproducible flat fracture surfaces. To reproduce these realistic fractures, patient CT data were segmented and converted into 3D-printed models of the fractured distal femur. From these, silicone molds were manufactured and subsequently filled with validated fluid polyurethane-based bone materials [23, 24], allowing a faithful replication of the interdigitated fracture morphology. This procedure ensured reproducibility across specimens while preserving the complexity of a patient-specific fracture pattern [25]. The mold-based manufacturing protocol ensured that the patient-derived interdigitated fracture morphology was reproduced consistently across all specimens, with sub-millimeter variation between samples [23, 24]. Osteotomies were performed using a custom 3D-printed cutting guide with a fixed saw slot on the identical synthetic femur geometry, ensuring a repeatable cut position and orientation. The fracture line was positioned 6 cm proximal to the intercondylar notch. The realistic fracture geometry (AO/OTA 33-A3) corresponds to the patient-derived model used in an earlier publication; for the present study, a new batch of specimens was produced using the same validated manufacturing protocol [25]. In contrast to the earlier work—which maintained a fixed diaphyseal screw configuration and included combined cyclic axial and torsional loading—the current protocol systematically varies working length by sequentially reducing shaft screws from five to two under isolated axial and torsional ramps. Four experimental groups (Fig. 1) were established: an osteotomized fracture with a 5 mm gap (OG), an osteotomized fracture with a closed gap (OC), a real fracture with a 5 mm gap (RG) and a real fracture with a closed gap (RC) [25].

All specimens (*n* = 8 per group) were stabilized with anatomically pre-contoured 7-hole distal femur locking compression plates (LCP DF, titanium alloy, DePuy Synthes Inc., Paoli, PA, USA). Fixation was performed by an experienced trauma surgeon according to the manufacturer’s surgical technique. Distally, 5 mm self-tapping locking screws of varying lengths (DePuy Synthes, Paoli, PA, USA) were inserted in holes A, C, D, E, F, and G. In the diaphyseal segment, screws of the same type and diameter were placed in holes 2 to 6. Working length was subsequently varied by sequential removal of shaft screws as described below.

To investigate the effect of working length, the five screws in the femoral shaft of the plate were sequentially removed, starting distally and progressing proximally, until only 2 screws were left. After each adjustment, mechanical tests were repeated on the same specimen to isolate the influence of working length.

### Mechanical testing

**Fig. 2:**
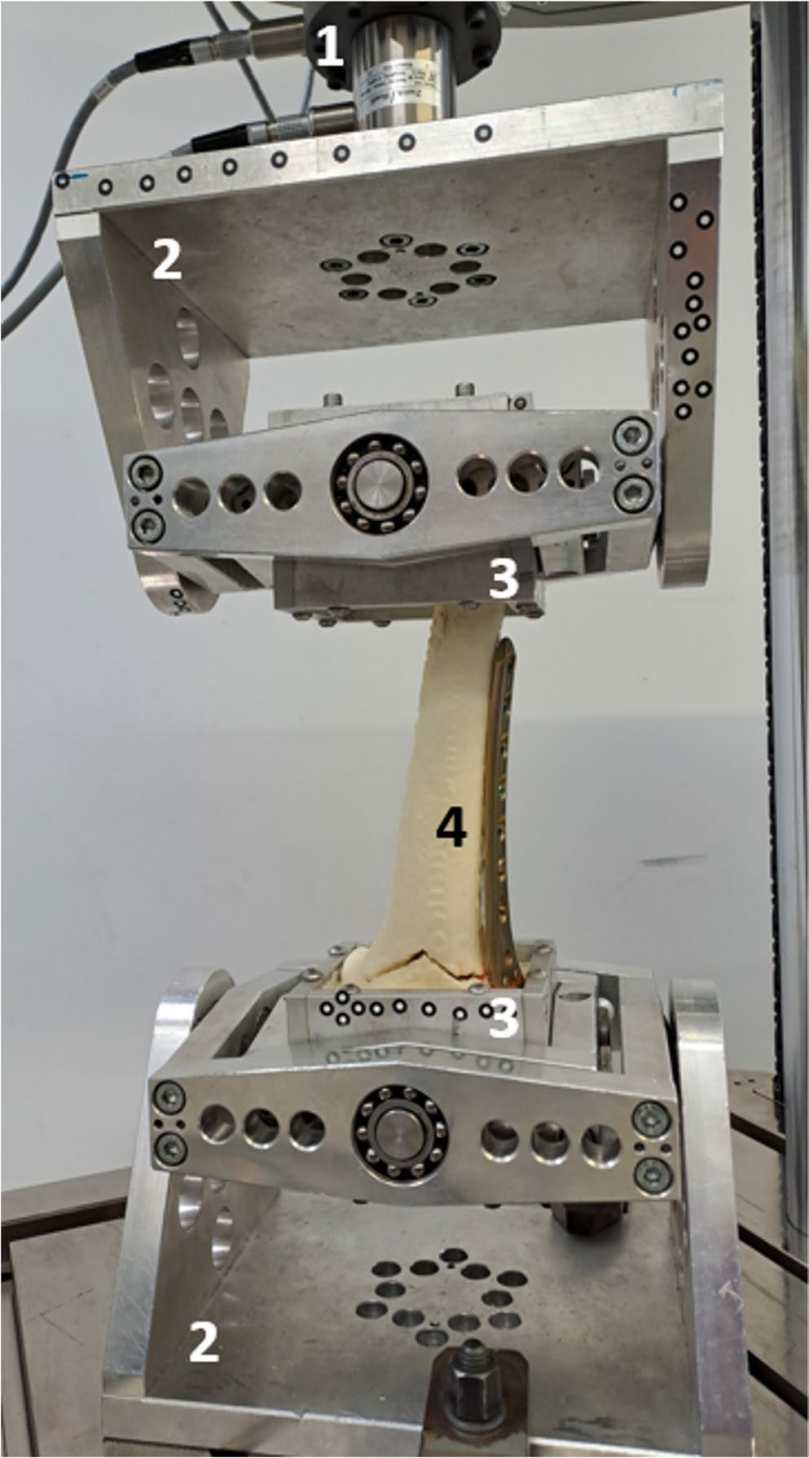
Test setup (1: force/torque actuator with load cell, 2: cardan joints, 3: embedding pots, 4: bone surrogate equipped with locking plate)

The distal and proximal bone segments were embedded in fast-curing resin (GP010, Gößl & Pfaff GmbH, Karlskron, Germany). To allow free implant movement during testing, screws and plates were covered with modeling clay (Noris Staedtler Mars GmbH & Co. Kg, Nürnberg, Germany). The distal femur was embedded at a physiological 6° valgus angle [26, 27] and secured in cardan joints proximally and distally [28] (LTM Z010, ZwickRoell GmbH & Co. KG, Ulm, Germany, Fig. 2).

Axial loading was performed at a ramp rate of 0.2 mm/s until 1500 N, and torsional loading was performed at 0.15°/s up to ± 4 Nm limiting the load within the linear elastic region [25]. During torsional loading, no axial preload or displacement was applied. The specimens were kept in physiological position, and the axial axis was fixed so that pure torsional loading was introduced without any additional axial force. Positive rotation corresponds to internal rotation of the femur, while negative rotation corresponds to external rotation. The axial and torsional stiffness of each construct was calculated from slopes of the load-deformation and angle-torque curves, respectively [25].

### Interfragmentary motion analysis

**Fig. 3:**
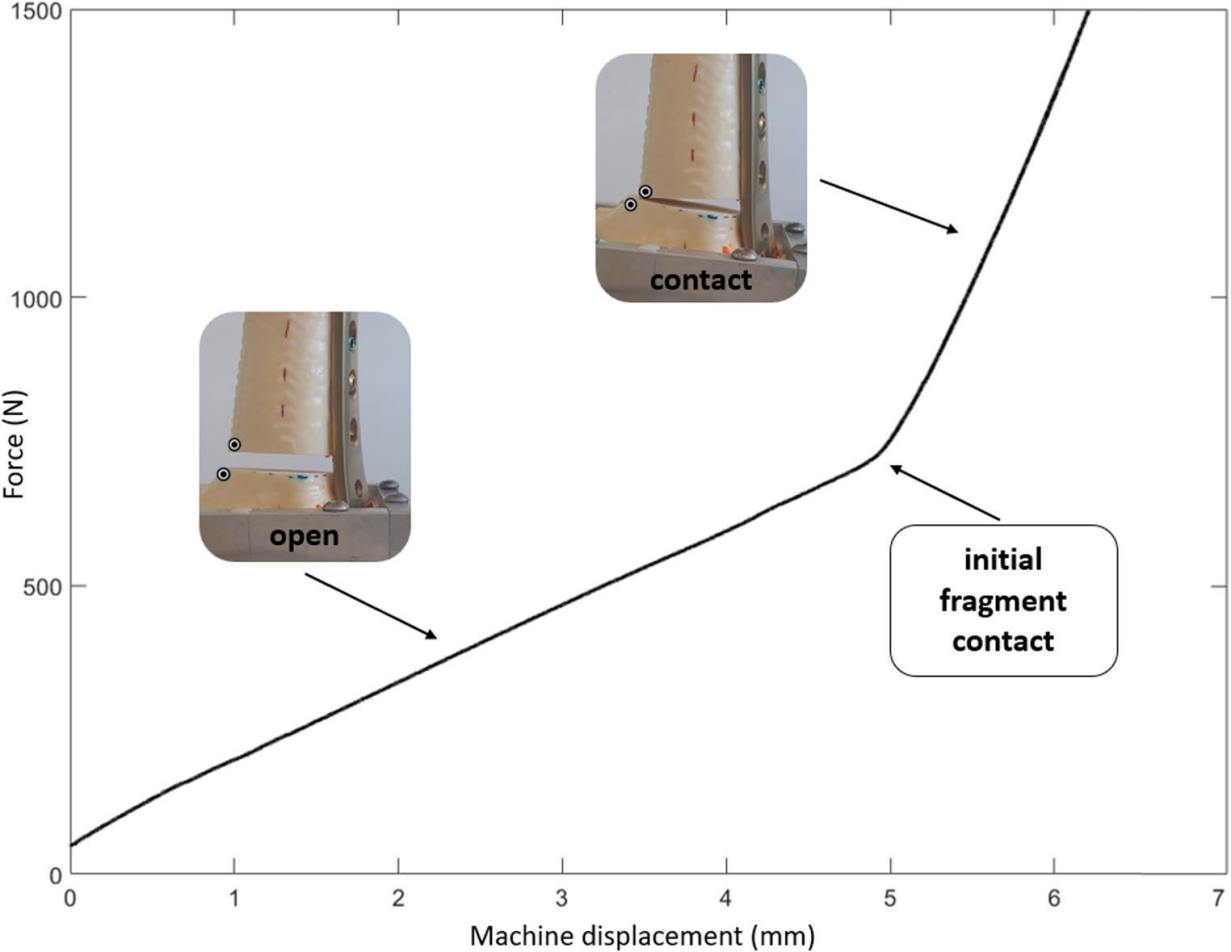
Force-displacement curve and construct states during axial loading. Inflection point indicates initial medial fragment contact. Images show OG constructs in the “open” and “contact” phases. Adhesive markers indicate shear displacement measurement point on the medial cortex. A comparable two-phase behavior (open/contact) was likewise observed in torque– rotation curves under torsional loading

Interfragmentary motions were evaluated using a 3D optical motion capture system (ARAMIS Professional 6 M, Zeiss GOM metrology GmbH, Braunschweig, Germany). Shear motion under axial and torsional loading were assessed using adhesive optical markers placed medially, directly adjacent to the fracture gap on both fragments. For each construct, local coordinate systems were established at anatomically corresponding points on either side of the fracture gap at the most medial location on the shaft (see adhesive marker points in Fig. 3). The amount of shear was analyzed as a vector quantity describing the relative displacement between the superior marker point in the axial plane with respect to the inferior marker point.

### Data analysis

Statistical analyses were performed using IBM SPSS Statistics version 26 (IBM Corp., Armonk, NY, USA). A mixed-design analysis of variance (ANOVA) was conducted to assess the effects of screw number and fracture type on axial and torsional stiffness. Mauchly’s test was used to assess the assumption of sphericity. In the event of a violation, the Greenhouse-Geisser correction was applied. Effect sizes were reported using partial eta squared (η²ₚ). The significance level was set at α = 0.05. No post-hoc analyses were conducted, as the primary focus was on overall main effects and interaction patterns.

## Results

### Axial construct stiffness

In two groups—OG and RG—two distinct phases were observed during axial loading. Initially, a 5 mm fracture gap was present and with increasing axial load, the gap closed and the bone fragments came into contact. Loading induced a bending of the plate and angular displacement across the fracture gap, resulting in initial fragment contact on the medial cortex, due to the lateral placement of the osteosynthesis plate. Consequently, while medial bone contact occurred, the 5 mm fracture gap on the lateral (plate) side was maintained. This transition was clearly identifiable in the force-displacement curves by a visible inflection point and a change in slope (Fig. 3). As a result, stiffness was evaluated separately for the “open” phase (before medial fragment contact, OGopen) and the “contact” phase (after medial fragment contact, OGcontact).

**Fig. 4:**
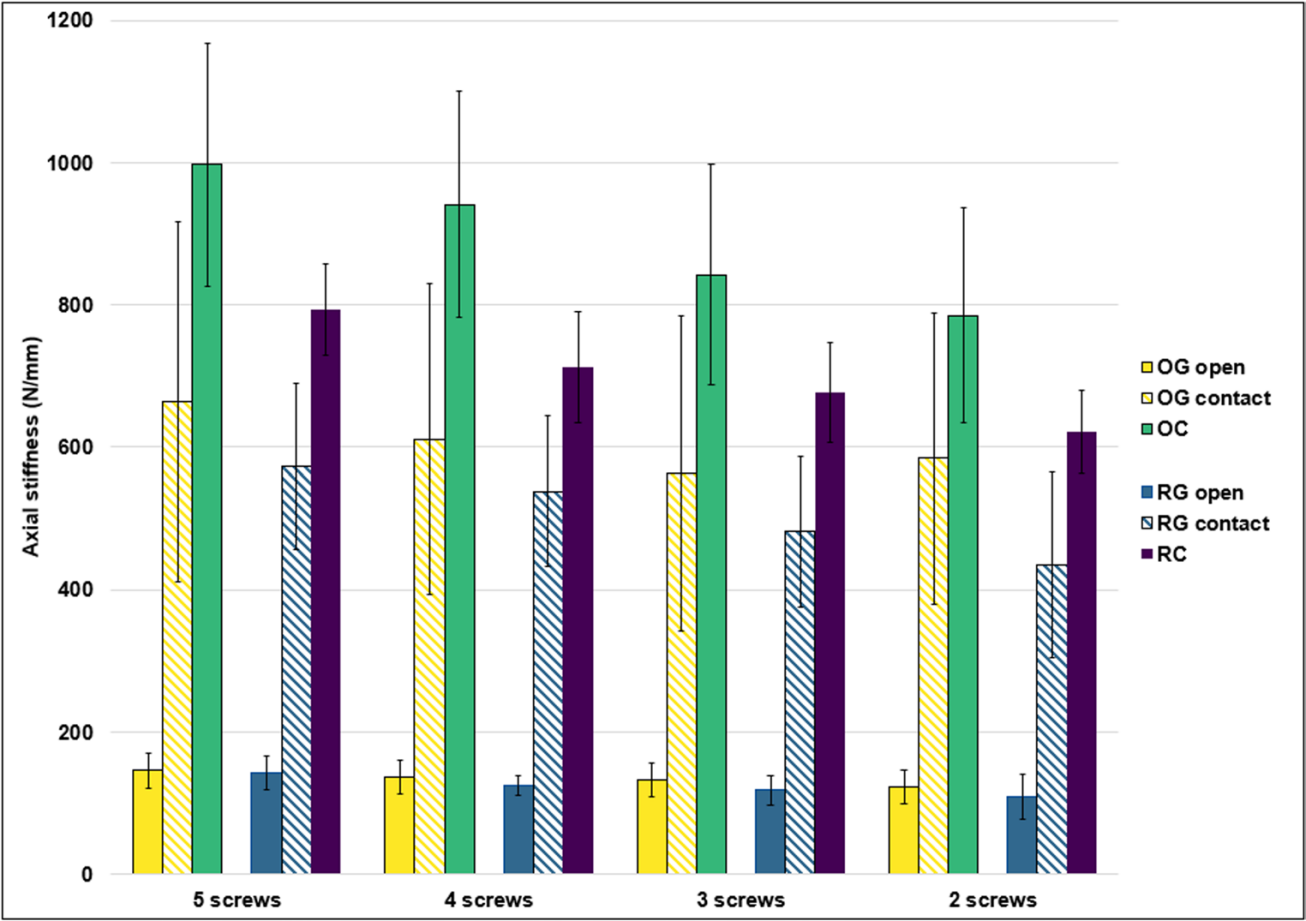
Mean axial stiffness of osteosynthesis constructs under axial loading, categorized by fracture types (OG, OC, RG, RC) and working lengths of the osteosynthesis plate (determined by screw configurations ranging from 2 to 5 screws). Constructions with a gap are further divided into stiffness before fracture closure (open) and after fracture closure (contact). Whiskers indicate standard deviations

Axial stiffness varied across the different groups and was influenced by fracture morphology, the presence of a gap, and the increase of working length due to progressive reduction of screws in the femoral shaft (Fig. 4).

In OG before gap closure (OGopen), increasing the working length decreased stiffness from 146 N/mm to 122 N/mm (−16%). After fracture closure (OGcontact), stiffness increased significantly to 663 N/mm with five screws before decreasing to 584 N/mm with two screws (12%). OC exhibited the highest stiffness values, starting at 997 N/mm with five screws and decreasing to 785 N/mm with two screws (−21%).

In RG before gap closure (RGopen), stiffness was initially 142 N/mm and dropped to 108 N/mm (−24%) with 2 screws. After fracture closure (RGcontact), stiffness increased to 573 N/mm with five screws, then declined to 435 N/mm with two screws (24%). RC showed higher initial stiffness than RG-groups, with 792 N/mm at five screws, reducing to 621 N/mm with two screws (−22%).

**Fig. 5:**
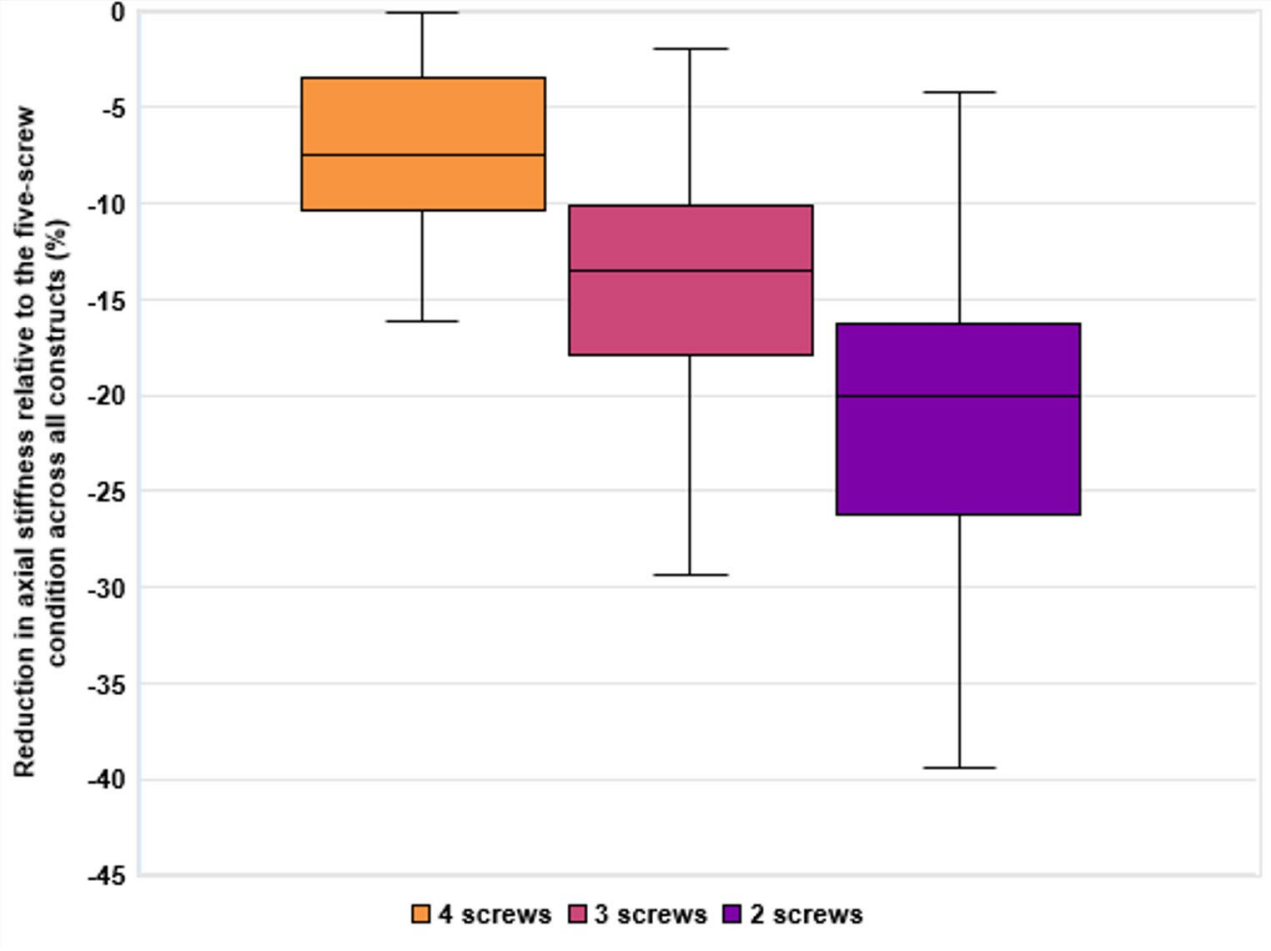
Reduction in axial stiffness relative to the five-screw condition across all constructs. Values are shown as mean percentage change (%), with 100% corresponding to the stiffness measured in each construct at full diaphyseal fixation (five screws). Boxes represent the interquartile range (25th to 75th percentile), with the median indicated by a horizontal line. Whiskers extend to the minimum and maximum values within 1.5 times the interquartile range

To visualize the overall reduction in axial stiffness with decreasing screw number, the percentage change from the five-screw condition was calculated for each fracture construct and summarized across all constructs at each screw level. Figure 5 displays the distribution of stiffness reduction values for 4, 3, and 2 screws, highlighting the progressive decrease in stiffness as screw count decreases, along with increasing variability across constructs.

To statistically assess the effects of construct type and screw number on axial stiffness, a mixed-design ANOVA was conducted. Mauchly’s test of sphericity was significant (W = 0.214, χ²(5) = 41.20, *p* < 0.001) indicating that the assumption of sphericity was violated. Therefore, results were reported using the Greenhouse-Geisser correction.

There was a significant main effect of fracture type (F(5, 42) = 32.36, *p* < 0.001, η²ₚ = 0.852) indicating overall differences in axial stiffness among the six fracture groups, regardless of screw number. Further, there was a significant main effect of screw number on axial stiffness, (F(1.61, 44.94) = 95.36, *p* < 0.001, η²ₚ = 0.773) indicating a strong effect: as the number of screws decreased, axial stiffness significantly declined. Additionally, there was a significant interaction between screw number and fracture type (F(8.03, 44.94) = 7.94, *p* < 0.001, η²ₚ = 0.587) suggesting that the effect of screw number on axial stiffness varied across the different fracture types.

### Torsional construct stiffness

**Fig. 6:**
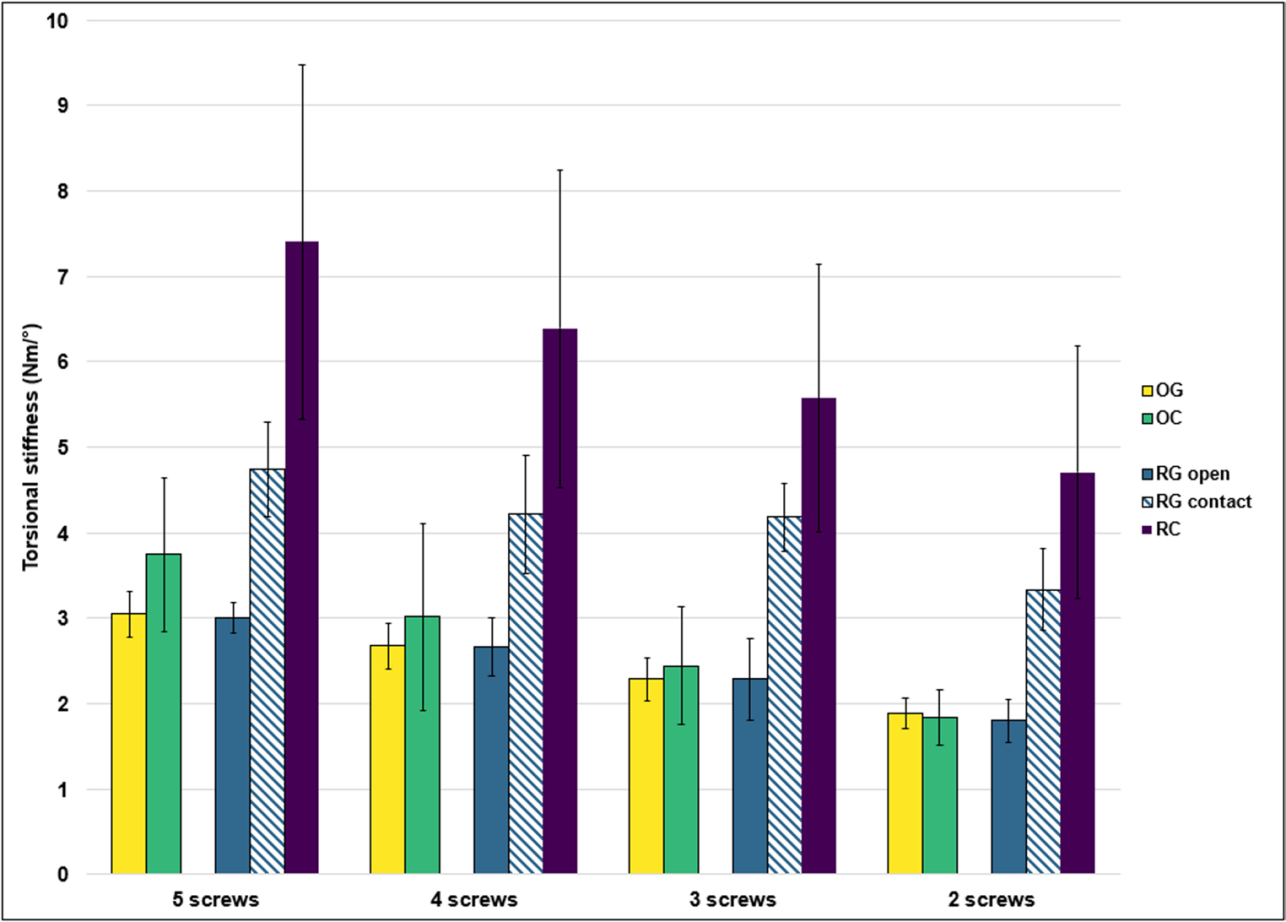
Mean external torsional stiffness of osteosynthesis constructs under torsional loading, categorized by fracture types (OG, CC, RG, RC) and working lengths of the osteosynthesis plate (determined by screw configurations ranging from 2 to 5 screws). Realistic fracture with gap (RG) was further divided into stiffness before fracture closure (open) and after fracture closure (contact). Whiskers represent standard deviations

Torsional stiffness varied across the groups and was influenced by fracture morphology, the presence of a gap, and the number of screws (Fig. 6). Similar to the axial loading tests in the OG and RG groups, where increasing load resulted in fracture gap closure and fragment contact, a comparable phenomenon was observed during torsional loading in the RG group (real fracture with gap). As external rotation progressed, the fracture fragments gradually came into contact. This transition was clearly reflected in the torque-rotation curves by a distinct increase in slope, indicating a higher torsional stiffness once contact was established. Therefore, torsional stiffness in the RG group was evaluated separately for the “open” phase (prior to fragment contact) and the “contact” phase (after contact had occurred). This contact-related increase in stiffness was not observed in the internal rotation direction, likely due to the asymmetrical orientation of the fracture lines in the realistic fracture model. The steeper configuration of the fracture surfaces in one direction promoted interlocking under external rotation, while in the internal direction the fragments remained unconstrained throughout the loading cycle.

In OG, the increase in working length decreased external rotation stiffness from 3.05 Nm/° to 1.88 Nm/° - (38%), while internal rotation stiffness declined from 2.84 Nm/° to 1.72 Nm/° (− 39%). In OC the increase in working length decreased external rotation stiffness from 3.75 Nm/° to 1.84 Nm/° (− 51%) and internal rotation stiffness from 3.32 Nm/° to 1.84 Nm/° (− 45%).

In RG, external rotation stiffness was evaluated separately for the open and contact phases. In the open phase (RGopen), stiffness decreased from 3.01 Nm/° to 1.80 Nm/° (−40%), while in the contact phase (RGcontact), it declined from 4.74 Nm/° to 3.37 Nm/° (−29%). Internal rotation stiffness decreased from 2.83 Nm/° to 1.69 Nm/° (−40%). RC exhibited the highest torsional stiffness, with external rotation dropping from 7.40 Nm/° to 4.71 Nm/° (−36%) and internal rotation from 5.84 Nm/° to 3.06 Nm/° (−48%).

**Fig. 7:**
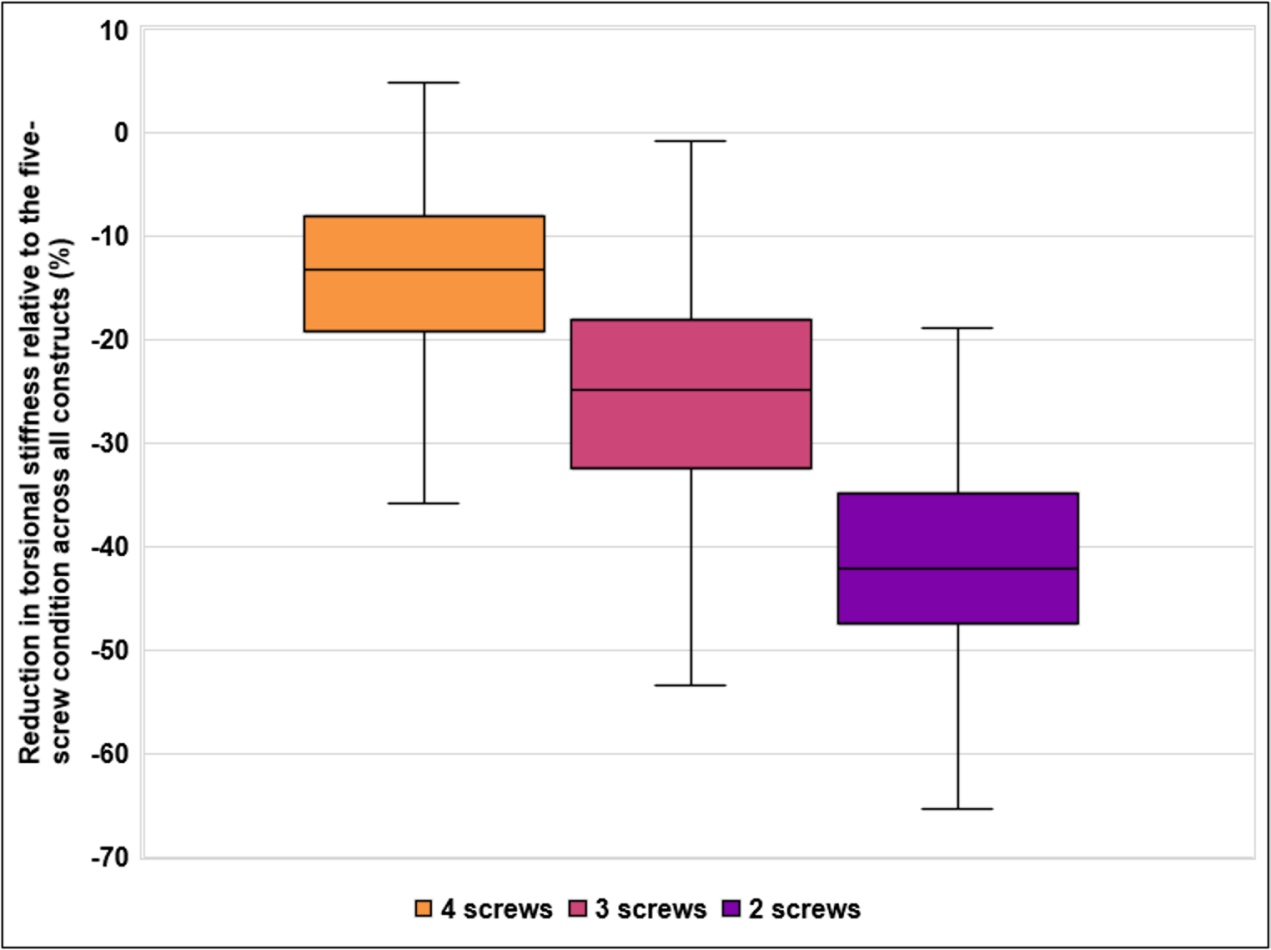
Reduction in torsional stiffness relative to the five-screw condition across all constructs. Values are shown as mean percentage change (%), with 100% corresponding to the stiffness measured in each construct at full diaphyseal fixation (five screws). Boxes represent the interquartile range (25th to 75th percentile), with the median indicated by a horizontal line. Whiskers extend to the minimum and maximum values within 1.5 times the interquartile range

To visualize the overall reduction in torsional stiffness with decreasing screw number, the percentage change from the five-screw condition was calculated for each construct and summarized across all constructs at each screw level. Figure 7 displays the distribution of torsional stiffness reduction for 4, 3, and 2 screws, showing a progressive decline in torsional stiffness across constructs with fewer screws.

These observed differences in torsional stiffness were statistically confirmed by repeated-measures analysis of variance. Mauchly’s test of sphericity was violated for the factor screw number (W = 0.065, χ²(5) = 53.77, *p* < 0.001). Therefore, Greenhouse-Geisser corrections were applied where appropriate.

There was a significant main effect of the fracture construct (F(3, 21) = 22.46, *p* < 0.001, η²ₚ = 0.762) indicating that torsional stiffness differed significantly between constructs. A significant main effect of screw number was observed (F(1.24, 26.04) = 8.01, *p* = 0.006, η²ₚ = 0.276) as well as a significant screw - fracture construct interaction (F(3.72, 26.04) = 11.27, *p* < 0.001, η²ₚ = 0.617) suggesting that the effect of screw reduction varied across constructs.

### Shear motion

**Fig. 8:**
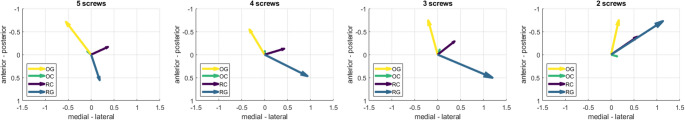
Resultant shear vectors under axial loading for all constructs across screw configurations. Vector direction and length represent the orientation and magnitude (in mm) of medial shear displacement

Under axial loading, the direction and magnitude of resultant shear vectors varied with construct type and screw configuration (Fig. 8). OC showed consistently small vectors, shifting from medial-posterior to lateral-anterior with reduced screw number. RC vectors remained posterior-lateral with minimal change in magnitude, reflecting constant stability due to fragment interlocking and absence of a gap. Constructs with fracture gaps (OG and RG) displayed larger and more variable shear displacements. OG shifted from medial-posterior to posterior with fewer screws. In the RG group, increasing working length was associated with a progression of shear vectors from anterolateral to posterolateral directions, accompanied by a rise in magnitude. This pattern indicates sliding along the anatomically realistic fracture surface, suggesting that specific fracture geometries may promote shear displacement between fragments.

**Fig. 9:**
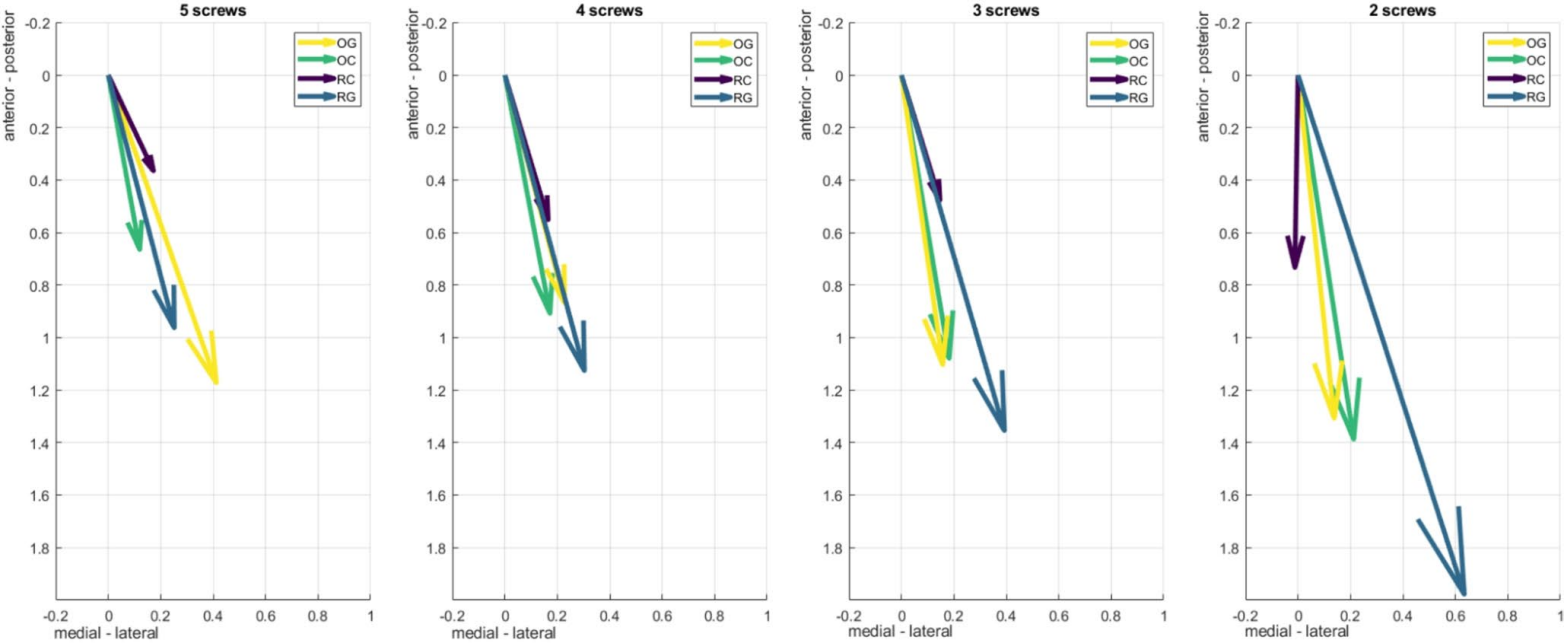
Resultant shear vectors under torsional loading for all constructs and screw configurations. Vector direction and length represent orientation and magnitude (in mm) of shear displacement during internal rotation

Under torsional loading, shear vector orientation was consistent across constructs (Fig. 9). External rotation produced medially-posterior displacement, while internal rotation resulted in lateral-anterior pointing vectors. RC showed the lowest shear displacement due to fracture interlocking, followed by OC. RG and OG had greater vector magnitudes, with RG showing the highest displacement from four screws onward.

## Discussion

This study examined how fracture morphology, fracture gap, and working length affect the mechanical behavior of osteosynthesis constructs under axial and torsional loading. A controlled synthetic femur model incorporating both osteotomized and realistic fractures was used to assess axial and torsional stiffness as well as interfragmentary shear motion across different screw configurations. The findings highlight the substantial influence of fracture type and fixation strategy on construct performance.

Fracture morphology emerged as a primary determinant of construct stiffness. OC demonstrated the highest axial stiffness, benefiting from uniform contact surfaces and reproducible geometry [8, 9]. Realistic fractures without gaps (RC) demonstrated lower stiffness than OC, but clearly outperformed constructs with a fracture gap, highlighting the stabilizing role of fragment contact and interlocking morphology [13, 29]. In contrast, both osteotomized (OG) and realistic (RG) fractures with a gap showed substantially reduced stiffness in the “open” phase, prior to fragment contact. At this stage, the osteosynthesis plate was the sole load-bearing structure, and the differences in fracture morphology were mechanically negligible. Upon progressive loading, fragment contact was established—first medially—leading to an increase in stiffness. In this “contact” phase, OG constructs were stiffer than RG constructs, likely due to the larger and more uniform contact surface created by the planar osteotomy. This illustrates that although realistic fractures may provide interlocking once in contact, their irregular morphology can limit full surface engagement, resulting in lower axial construct stiffness compared to idealized osteotomies.

A unique aspect of this study was the evaluation of the “open” and “contact” states in constructs with initial fracture gaps [30, 31]. In OG and RG groups under axial loading, stiffness was initially low in the open phase due to the absence of fragment contact—reflecting the bending resistance of the plate alone. With increasing load, the plate deflected and brought the medial cortices into contact, leading to a distinct increase in stiffness in the contact phase. However, this increase did not reach the levels observed in OC, where direct cortical contact existed from the beginning. The reduced axial stiffness in the contact phase of OG and RG can be attributed to the fact that only the medial edges of the fragments touched, and the proximal cortex pinched the cancellous bone of the distal fragment without full cortical interlock. While OG contact stiffness increased due to uniform cortical compression, RG contact benefited from morphological interlocking, but only partially and asymmetrically. This difference illustrates how osteotomized models can overestimate axial stability under idealized contact conditions, whereas real fractures display more complex, direction-dependent mechanical behavior.

Torsional testing also revealed direction-dependent contact phenomena in the RG group. Due to the specific geometry of the realistic fracture surface, fragment contact within the tested range occurred only in one rotation direction. Once contact was established during loading, torsional stiffness increased, reflecting a transition from unconstrained to interlocked fragment behavior. This asymmetry was absent in osteotomized models, further emphasizing the mechanical relevance of realistic fracture morphology. This may reflect intrinsic asymmetries in load transfer and fragment mobility due to fracture morphology.

The number of screws in the diaphyseal segment, and consequently the working length, significantly influenced construct behavior. A systematic reduction in screw number led to decreased axial and torsional stiffness across all groups. However, the magnitude of stiffness loss varied by fracture type, suggesting that working length interacts with fracture morphology. Constructs with gaps were more sensitive to screw reduction, as indicated by larger relative declines in stiffness and greater variability in response [1, 3]. A shorter working length in plate fixation constructs generally increases overall construct stiffness and reduces plate strain, as demonstrated in multiple studies [2, 3, 32]. However, the effect is not universal. MacLeod et al. showed that in small-gap or well-reduced constructs, shorter working lengths may actually increase plate stress [33]. Thus, while the prevailing evidence supports shorter working lengths as stiffer configurations with reduced plate strain, the interaction with fracture morphology, gap size, and loading conditions must be considered.

This interaction is further illustrated by the boxplot analysis across all constructs. Although fracture morphology and gap size played a significant role in absolute stiffness values, the percentage reduction in stiffness relative to the five-screw conditions demonstrated a consistent pattern: fewer screws resulted in greater stiffness loss. This trend was observed both under axial and torsional loading and reflects the critical influence of working length on construct mechanics. The increasing variability at lower screw counts further supports the importance of secure fixation in maintaining consistent mechanical behavior. This visual trend is supported by the statistical analysis, which showed large effect sizes for screw number in both axial (η²ₚ = 0.773) and torsional loading (η²ₚ = 0.617), underscoring the critical role of working length in overall construct behavior.

Under axial loading, resultant shear vectors lengthened with each reduction in screw number, indicating increased micromotion in the fracture zone. The smallest shear displacements were observed in both OC and RC constructs, reflecting high stability due to direct fragment contact—whether planar or interdigitated. In contrast, RG constructs exhibited the largest shear motions, particularly at reduced screw numbers, highlighting the susceptibility of realistic fracture gaps to displacement. Notably, the direction of shear vectors also shifted with fracture morphology, despite identical embedding and loading conditions. This underscores that fracture geometry alone can alter both the magnitude and path of micromotion under standardized test setups. The resultant vectors further confirmed that increased working length consistently led to increased interfragmentary motion across all groups.

Under torsional loading, consistent directional differences emerged between internal and external rotation, particularly in constructs with gaps. RC showed the smallest shear motions, followed by OC. RG and OG exhibited larger displacements, with RG most affected by screw reduction. The steep fracture line and delayed fragment contact in RG contributed to a sudden increase in shear displacement once the fragments began to slide post-contact. These mechanical responses illustrate the compounding effect of gap, morphology, and working length on construct behavior.

The use of synthetic bone models represents a methodological limitation, as such models lack the biological heterogeneity of human bone tissue [23, 24]. Moreover, only a single real fracture morphology was imprinted into the synthetic bone. Other fracture types—or variations in fracture height and orientation—might lead to different contact behaviors or stiffness patterns. This directional effect was evident in the RG group, where contact occurred only during external rotation; with a different geometry, such asymmetry may be reduced or reversed. However, synthetic models offer the advantage of controlled and reproducible fracture morphology.

Unlike in human donor bone, realistic fractures can be consistently integrated into synthetic models using our standardized manufacturing protocol, which is essential for consistent and comparative mechanical testing. The results are specific to the patient-derived interdigitated fracture morphology tested. Other fracture types, particularly multi-fragmentary or oblique configurations, may lead to different contact mechanics and construct behavior. Moreover, muscular forces were not simulated in the current setup. In vivo, gastrocnemius and quadriceps traction alter joint reaction forces and may interact with fracture morphology and working length. Future studies should therefore integrate muscle-force simulation and additional fracture geometries to validate the generalizability of the present results.

These findings underscore the limitations of using osteotomized fractures in biomechanical testing, particularly when assessing torsional behavior or shear motion. While osteotomies allow for standardized and reproducible testing [8, 10], they do not adequately capture the stabilizing effects of real fracture morphology. For studies involving rotational loading or fragment micromotion, the use of realistic fracture models is recommended to ensure clinically relevant insights [34, 35]. Clinical relevance in this context refers to the fact that fixation stability in everyday practice is often determined by irregular fracture morphologies, comminution, or residual gaps, which are not represented in simplified osteotomy models. By incorporating these clinically encountered fracture characteristics, biomechanical studies can better inform implant choice, screw configuration, and the need for supplemental fixation in complex distal femur fractures.

From a clinical perspective, the results support the importance of anatomical reduction to maximize construct stability, as contact conditions increased axial and torsional stiffness by approximately 30–40% compared to constructs with fracture gaps. Realistic fractures benefit from interdigitated contact surfaces, but only when fragment contact is achieved. Fixation strategies should consider both fracture type and working length. Shorter working lengths improve construct stiffness but may reduce flexibility needed for secondary bone healing [1, 4]. Therefore, implant configurations should balance mechanical stability with biological healing potential, especially in fractures prone to micromotion. In clinical practice, distal femur fractures—particularly comminuted patterns—frequently present with residual fracture gaps. Based on our findings, short working lengths may offer mechanical advantages in such cases by reducing shear motion and enhancing initial construct stiffness, thereby minimizing the risk of early displacement. However, this comes at the expense of reduced flexibility, which could be detrimental to secondary bone healing, and in combination with good reduction may also increase plate stress, as reported by MacLeod et al. [33]. Clinical decision-making should therefore balance mechanical stabilization with the biological requirements for callus formation. The observed medial fragment contact during loading—due to the lateral plate position—suggests a potential for varus collapse or axis deviation in fractures lacking medial cortical support. In such cases, the addition of a medial plate may be considered to prevent asymmetric loading and maintain alignment during early weight-bearing.

An important advantage of this standardized manufacturing protocol is that once a patient-derived fracture morphology is segmented and transferred into a mold, it can be reproduced consistently in any number of synthetic specimens. This allows other research groups or implant manufacturers to investigate identical fracture geometries under varying implant designs or loading conditions, thereby improving comparability and reproducibility across biomechanical studies. Although this study was conducted using synthetic bones, the fracture morphology was patient-specific and replicable, supporting its relevance. Transferability to cadaveric models is feasible, and future validation under more physiological conditions—particularly with soft tissue and muscle force simulation—would be a logical next step to confirm the biomechanical implications observed here. A limitation of this study is that only isolated axial and torsional loads were applied. These loading modes do not represent the full complexity of physiological conditions, which are multidirectional and strongly influenced by soft tissues and muscle forces. However, the use of simplified, reproducible test conditions enabled an isolation of the effect of fracture morphology itself. Within these standardized scenarios, realistic fractures exhibited distinct mechanical behaviors (e.g., asymmetric interlocking under torsion, medial cortical contact under axial load) that were not present in osteotomized models. This highlights the specific role of fracture morphology while acknowledging that future studies should incorporate combined, cyclic, and muscle-force–driven loading to further enhance clinical relevance.

## Conclusion

This study highlights the fundamental biomechanical differences between osteotomized and real fractures, particularly in terms of torsional and axial stability. Real fractures without a gap (RC) exhibited the highest torsional stiffness among all groups, while osteotomized fractures with a gap (OG) had the lowest, reinforcing the importance of fracture morphology in rotational stability. Conversely, osteotomized fractures demonstrated greater axial stiffness due to their smooth, planar surfaces facilitating interfragmentary compression. The number of screws and resulting working length further modulated construct behavior across all groups, with fewer screws leading to reduced stiffness and increased interfragmentary motion. These findings emphasize the necessity of anatomical reduction and fixation planning in clinical fracture management and suggest that experimental studies investigating torsional stability should prioritize real fracture models to ensure clinically relevant results.

## Data Availability

The raw data can be obtained on request from the corresponding author.

## References

[1] Chao P, Conrad BP, Lewis DD, Horodyski M, Pozzi A. Effect of plate working length on plate stiffness and cyclic fatigue life in a cadaveric femoral fracture gap model stabilized with a 12-hole 2.4 mm locking compression plate. BMC Vet Res. 2013;9:125. 10.1186/1746-6148-9-125. 23800317 10.1186/1746-6148-9-125 PMC3704939

[2] Trefny FN, Glyde MR, Hosgood GL, Day RE, Hayes A. Effect of plate screw configuration on construct stiffness and plate strain in a synthetic short fragment small gap fracture model stabilized with a 12-hole 3.5-mm locking compression plate. Vet Comp Orthop Traumatol. 2025;38(3):119–26. 10.1055/s-0044-1791701. 39366420 10.1055/s-0044-1791701

[3] Wainberg SH, Moens NMM, Ouyang Z, Runcoman J. The effect of working length, fracture, and screw configuration on plate strain in a 3.5-mm LCP bone model of comminuted fractures. VCOT Open. 2023;06(02):e122-35.

[4] Kim TH, et al. Fracture gap and working length are important actionable factors affecting bone union after minimally invasive plate osteosynthesis for the treatment of simple diaphyseal or distal metaphyseal tibia fractures. Orthop Traumatol Surg Res. 2024;110(2):103770. 10.1016/j.otsr.2023.103770. 37979671 10.1016/j.otsr.2023.103770

[5] Mardian S, Schaser KD, Duda GN, Heyland M. Working length of locking plates determines interfragmentary movement in distal femur fractures under physiological loading. Clin Biomech (Bristol). 2015;30(4):391–6. 10.1016/j.clinbiomech.2015.02.006. 25716162 10.1016/j.clinbiomech.2015.02.006

[6] Augat P, Burger J, Schorlemmer S, Henke T, Peraus M, Claes L. Shear movement at the fracture site delays healing in a diaphyseal fracture model. J Orthop Res. 2003;21(6):1011–7. 10.1016/S0736-0266(03)00098-6. 14554213 10.1016/S0736-0266(03)00098-6

[7] Augat P, Hollensteiner M, von Ruden C. The role of mechanical stimulation in the enhancement of bone healing. Injury. 2021; 52(Suppl 2): S78-S83. 10.1016/j.injury.2020.10.009 10.1016/j.injury.2020.10.009 33041020

[8] de Bruyn BW, Glyde M, Day R, Hosgood G. Effect of an orthogonal locking plate and primary plate working length on construct stiffness and plate strain in an in vitro fracture-gap model. Vet Comp Orthop Traumatol. 2024;37(4):173–80. 10.1055/s-0044-1779496. 38331034 10.1055/s-0044-1779496

[9] Evans A, Glyde M, Day R, Hosgood G. Effect of plate-bone distance and working length on 2.0-mm locking construct stiffness and plate strain in a diaphyseal fracture gap model: a biomechanical study. Vet Comp Orthop Traumatol. 2024;37(1):1–7. 10.1055/s-0043-1771198. 37473771 10.1055/s-0043-1771198

[10] Hoffmeier KL, Hofmann GO, Muckley T. Choosing a proper working length can improve the lifespan of locked plates. A biomechanical study. Clin Biomech (Bristol). 2011;26(4):405–9. 10.1016/j.clinbiomech.2010.11.020. 21185629 10.1016/j.clinbiomech.2010.11.020

[11] Choudry RC. K, Biomechanics of Distal Femur Fractures. In: Kulkarni GS, Babhulkar S, editors. 2017. 10.1055/b-0042-186849.

[12] Nauth A, et al. Distal femur fractures: basic science and international perspectives. OTA International. 2024;7(2 Suppl):e320. 10.1097/OI9.0000000000000320. 38487402 10.1097/OI9.0000000000000320 PMC10936154

[13] Klein M, et al. Comparison of healing process in open osteotomy model and open fracture model: delayed healing of osteotomies after intramedullary screw fixation. J Orthop Res. 2015;33(7):971–8. 10.1002/jor.22861. 25732349 10.1002/jor.22861

[14] Meeson R, Moazen M, Sanghani-Kerai A, Osagie-Clouard L, Coathup M, Blunn G. The influence of gap size on the development of fracture union with a micro external fixator. J Mech Behav Biomed Mater. 2019;99:161–8. 10.1016/j.jmbbm.2019.07.015. 31357063 10.1016/j.jmbbm.2019.07.015 PMC6715773

[15] Wazen RM, Currey JA, Guo H, Brunski JB, Helms JA, Nanci A. Micromotion-induced strain fields influence early stages of repair at bone-implant interfaces. Acta Biomater. 2013;9(5):6663–74. 10.1016/j.actbio.2013.01.014. 23337705 10.1016/j.actbio.2013.01.014 PMC3622828

[16] Bergmann G, et al. Standardized loads acting in knee implants. PLoS ONE. 2014;9(1):e86035. 10.1371/journal.pone.0086035. 24465856 10.1371/journal.pone.0086035 PMC3900456

[17] Harvin WH, et al. Working length and proximal screw constructs in plate osteosynthesis of distal femur fractures. Injury. 2017;48(11):2597–601. 10.1016/j.injury.2017.08.064. 28889934 10.1016/j.injury.2017.08.064

[18] MacLeod AR, Pankaj P. Pre-operative planning for fracture fixation using locking plates: device configuration and other considerations. Injury. 2018;49(Suppl 1):S12-S18. 10.1016/S0020-1383(18)30296-1 10.1016/S0020-1383(18)30296-1 29929685

[19] Claes LE, Heigele CA. Magnitudes of local stress and strain along bony surfaces predict the course and type of fracture healing. J Biomech. 1999;32(3):255–66. 10.1016/s0021-9290(98)00153-5. 10093025 10.1016/s0021-9290(98)00153-5

[20] Perren SM. Evolution of the internal fixation of long bone fractures. The scientific basis of biological internal fixation: choosing a new balance between stability and biology. J Bone Joint Surg Br. 2002; 84(8):1093 – 110. 10.1302/0301-620x.84b8.13752 10.1302/0301-620x.84b8.13752 12463652

[21] Fulkerson E, Egol KA, Kubiak EN, Liporace F, Kummer FJ, Koval KJ. Fixation of diaphyseal fractures with a segmental defect: a biomechanical comparison of locked and conventional plating techniques. J Trauma. 2006;60(4):830–5. 10.1097/01.ta.0000195462.53525.0c. 16612304 10.1097/01.ta.0000195462.53525.0c

[22] Kregor PJ, Stannard J, Zlowodzki M, Cole PA, Alonso J. Distal femoral fracture fixation utilizing the Less Invasive Stabilization System (L.I.S.S.): the technique and early results. Injury. 2001;32(Suppl 3):SC32-47. 10.1016/s0020-1383(01)00182-6 10.1016/s0020-1383(01)00182-6 11888193

[23] Hollensteiner M, et al. Population-specific femur models: a step towards improved osteosynthetic biomechanical testing in orthopaedics. Clin Biomech. 2025;121:106379. 10.1016/j.clinbiomech.2024.106379. 10.1016/j.clinbiomech.2024.106379 39550926

[24] Hollensteiner M, et al. Biomechanical validation of novel polyurethane-resin synthetic osteoporotic femoral bones in axial compression, four-point bending and torsion. Med Eng Phys. 2024;130:104210. 10.1016/j.medengphy.2024.104210. 39160032 10.1016/j.medengphy.2024.104210

[25] Hollensteiner M, et al. Impact of fracture morphology on the biomechanical stability of osteosynthetic fixation. Eur J Trauma Emerg Surg. 2025;51(1):144. 40111437 10.1007/s00068-025-02802-0 PMC11925963

[26] Mehling I, Hoehle P, Sternstein W, Blum J, Rommens PM. Nailing versus plating for comminuted fractures of the distal femur: a comparative biomechanical in vitro study of three implants. Eur J Trauma Emerg Surg. 2013;39(2):139–46. 10.1007/s00068-012-0247-1. 26815070 10.1007/s00068-012-0247-1

[27] Pietsch M, Hochegger M, Winkler M, Sandriesser S, Freude T, Augat P. Opening-wedge osteotomies of the distal femur: minor advantages for a biplanar compared to a uniplanar technique. Knee Surg Sports Traumatol Arthrosc. 2019;27(7):2375–84. 10.1007/s00167-018-5332-5. 30547307 10.1007/s00167-018-5332-5

[28] Schmidt U, Penzkofer R, Bachmaier S, Augat P. Implant material and design alter construct stiffness in distal femur locking plate fixation: a pilot study. Clin Orthop Relat Res. 2013;471(9):2808–14. 10.1007/s11999-013-2867-0. 23436162 10.1007/s11999-013-2867-0 PMC3734410

[29] Beerekamp MS, Haverlag R, Ubbink DT, Luitse JS, Ponsen KJ, Goslings JC. How to evaluate the quality of fracture reduction and fixation of the wrist and ankle in clinical practice: a delphi consensus. Arch Orthop Trauma Surg. 2011;131:739–46. 10.1007/s00402-010-1198-9. 20967547 10.1007/s00402-010-1198-9 PMC3095796

[30] Bottlang M, Doornink J, Fitzpatrick DC, Madey SM. Far cortical locking can reduce stiffness of locked plating constructs while retaining construct strength. J Bone Joint Surg Am. 2009;91(8):1985–94. 10.2106/JBJS.H.01038. 19651958 10.2106/JBJS.H.01038 PMC2714811

[31] Moazen M, et al. The effect of fracture stability on the performance of locking plate fixation in periprosthetic femoral fractures. J Arthroplasty. 2013;28(9):1589–95. 10.1016/j.arth.2013.03.022. 23642449 10.1016/j.arth.2013.03.022

[32] Stoffel K, Dieter U, Stachowiak G, Gachter A, Kuster MS. Biomechanical testing of the LCP–how can stability in locked internal fixators be controlled? Injury. 2003;34(Suppl 2):B11-9. 10.1016/j.injury.2003.09.021 10.1016/j.injury.2003.09.021 14580982

[33] MacLeod A, Simpson A, Pankaj P. Experimental and numerical investigation into the influence of loading conditions in biomechanical testing of locking plate fracture fixation devices. Bone Joint Res. 2018;7(1):111–20. 10.1302/2046-3758.71.BJR-2017-0074.R2. 29363522 10.1302/2046-3758.71.BJR-2017-0074.R2 PMC5805837

[34] Babst R, Beeres FJP, Link BC. Definitions and explanations on the topic of fracture reduction [Definitionen und Erklarungen zum Thema Frakturreposition]. Unfallchirurg. 2019;122(2):88–94. 10.1007/s00113-018-0573-9. 10.1007/s00113-018-0573-9 30402688

[35] Schmoelz W, Zierleyn JP, Hoermann R, Arora R. Standardized fracture creation in the distal humerus and the olecranon for surgical training and biomechanical testing. Arch Orthop Trauma Surg. 2022;142(12):3853–61. 10.1007/s00402-021-04286-0. 34973090 10.1007/s00402-021-04286-0 PMC9596540

